# Mitochondrial dysfunction in myofibrillar myopathy

**DOI:** 10.1016/j.nmd.2016.08.004

**Published:** 2016-10

**Authors:** Amy E. Vincent, John P. Grady, Mariana C. Rocha, Charlotte L. Alston, Karolina A. Rygiel, Rita Barresi, Robert W. Taylor, Doug M. Turnbull

**Affiliations:** a Wellcome Trust Centre for Mitochondrial Research, Institute of Neuroscience, Newcastle University, Newcastle upon Tyne, NE2 4HH, UK; b Rare Diseases Advisory Group Service for Neuromuscular Diseases, Muscle Immunoanalysis Unit, Newcastle upon Tyne Hospitals NHS Foundation Trust, Newcastle upon Tyne, NE2 4AZ, UK

**Keywords:** Myofibrillar myopathy, Mitochondria, Mitochondrial DNA deletion, Immunofluorescence, Histochemistry, Cytochrome *c* oxidase deficiency

## Abstract

Myofibrillar myopathies (MFM) are characterised by focal myofibrillar destruction and accumulation of myofibrillar elements as protein aggregates. They are caused by mutations in the *DES*, *MYOT*, *CRYAB*, *FLNC*, *BAG3*, *DNAJB6* and *ZASP* genes as well as other as yet unidentified genes. Previous studies have reported changes in mitochondrial morphology and cellular positioning, as well as clonally-expanded, large-scale mitochondrial DNA (mtDNA) deletions and focal respiratory chain deficiency in muscle of MFM patients. Here we examine skeletal muscle from patients with desmin (n = 6), ZASP (n = 1) and myotilin (n = 2) mutations and MFM protein aggregates, to understand how mitochondrial dysfunction may contribute to the underlying mechanisms causing disease pathology. We have used a validated quantitative immunofluorescent assay to study respiratory chain protein levels, together with oxidative enzyme histochemistry and single cell mitochondrial DNA analysis, to examine mitochondrial changes. Results demonstrate a small number of clonally-expanded mitochondrial DNA deletions, which we conclude are due to both ageing and disease pathology. Further to this we report higher levels of respiratory chain complex I and IV deficiency compared to age matched controls, although overall levels of respiratory deficient muscle fibres in patient biopsies are low. More strikingly, a significantly higher percentage of myofibrillar myopathy patient muscle fibres have a low mitochondrial mass compared to controls. We concluded this is mechanistically unrelated to desmin and myotilin protein aggregates; however, correlation between mitochondrial mass and muscle fibre area is found. We suggest this may be due to reduced mitochondrial biogenesis in combination with muscle fibre hypertrophy.

## Highlights

• Clonally expanded mtDNA deletions were found in a small number of patient fibres.
• Complex I and IV deficiency is higher than in control muscle.
• Mitochondrial mass is significantly reduced in patients relative to controls.
• No relationship between MFM protein aggregates and reduced mitochondrial mass.
• Negative correlations was detected between mitochondrial mass and muscle fibre area.

## 1 Introduction

Myofibrillar myopathies (MFMs) are a group of myopathies characterised by aggregation of the Z-disk proteins and focal myofibrillar destruction. MFMs or Z-disk diseases are clinically and genetically heterogeneous. Mutations causing MFM can be found in genes encoding a range of Z-disk proteins including; desmin (*DES*) [1], αB-crystallin (*CRYAB*) [2], myotilin (*MYOT*) [3], filamin C (*FLNC*) [4], ZASP (*LDB3/ZASP*) [5] and Bcl2-associated athanogene 3 (*BAG3*) [6]. Novel MFM causing genes are continuously being identified and at present only 50% of suspected MFM cases have a genetic diagnosis.

They are typically present in the third or fourth decade of life or later, though rare cases of adolescent onset have also been reported. Most commonly they present as distal myopathies initially, progressing to involve proximal limb muscles. However, myofibrillar myopathies can vary in clinical presentation and can lead to cardiomyopathy associated with skeletal muscle myopathy or isolated cardiomyopathy. Key clinical patterns in presentation can be noted for some of the causative genes; however, once more, these are not clear cut and there are exceptions.

Mitochondrial dysfunction is a common finding in many proteinopathies including protein aggregate myopathies (PAMs) [7], [8], [9], [10], [11], [12], [13], [14], [15], and some neurodegenerative conditions [16]. Cytochrome *c* oxidase (COX) deficiency has been found to be associated with clonally-expanded mtDNA rearrangements in sarcopenia, patients with single or multiple mitochondrial DNA (mtDNA) deletions and inclusion body myositis. However, mtDNA point mutations [17] and depletion of mtDNA copy number [18] may also lead to focal respiratory chain deficiency.

Previous reports have noted that both mitochondrial morphology and positioning are altered in muscle of MFM patients [19], as well as the presence of clonally-expanded large-scale mtDNA deletions [20], [21] and focal respiratory chain deficiency [22], [23]. However, there have only been a few previous publications to date and only small numbers of patients reported in these studies. As such it is unclear what role, if any, clonally expanded mtDNA deletion and mitochondrial respiratory chain deficiency may play in the pathogenesis of myofibrillar myopathy.

In this paper we investigate a cohort of nine genetically determined MFM patients for evidence of mitochondrial dysfunction. From this cohort, we further selected a group of six patients based on the frequency of COX-deficient fibres identified by sequential COX/SDH histochemistry to study for presence of clonally-expanded large-scale mtDNA deletions. We also subjected all patient muscle biopsies to a recently developed immunofluorescence assay [24] to assess NDUFB8 (complex-I) and COX-I (complex-IV) protein levels normalised to mitochondrial mass (Porin).

## 2 Materials and methods

### 2.1 Patient cohort

Muscle biopsies were taken for diagnostic purposes from patients with a suspected neuromuscular condition (see Table 1). Informed consent was obtained from all patients in the study. Ethical approval was granted by the Newcastle and North Tyneside local research and ethics committee (LREC2002/205). Inclusion criteria were the presence of protein aggregates on sections stained with anti-desmin and anti-myotilin antibodies and confirmed molecular diagnosis for MFM genes. We studied nine MFM patients with mutations in *DES* (n = 6), *MYOT* (n = 2) and *ZASP* (n = 1). A patient with compound heterozygous *RRM2B* variants, leading to a disturbance of mtDNA maintenance and multiple mtDNA deletions [25], was used as a positive control, as well as two healthy control muscle biopsies from individuals aged 52 and 63 years.

### 2.2 Histochemistry

Muscle was cryosectioned (10 µm) from transversely-orientated muscle blocks and subjected to histochemical reactions for the individual activities of COX, SDH and the sequential assay of COX/SDH activity [26]. COX-deficient fibres were counted using a stereomicroscope, by outlining the full muscle biopsy section and using the meander scan function in Stereo Investigator. Fibres were categorised by eye with blue fibres being categorised as COX negative, blue-grey fibres as intermediate negative and grey-brown fibres as intermediate positive. Intermediate categories were included to make comparison with immunofluorescent quantification easier. The minimum number of fibres counted was 127, which was for patient DES3 who had the smallest muscle biopsy section. Serial sections were stained with Haematoxylin and Eosin (H&E) to examine muscle morphology.

### 2.3 Quadruple immunofluorescence

A quadruple immunofluorescent method using antibodies recognising laminin (membrane marker), NDUFB8 (complex I marker), COX-I (complex IV marker), and porin as a mitochondrial mass marker, was employed to assess the degree of respiratory chain deficiency [24]. Briefly, one 10 µm cryosection from each patient and age matched controls was labelled with all four antibodies and a serial 10 µm section with just laminin to be used as a negative (no primary antibody) control. Sections were incubated with secondary antibodies coupled with Alexa Fluor 405, 488, 546 and 647 (Life Technologies). Using a Zeiss AxioImager and AxioCam MRm with AxioVision software tiled images scanning of the full section were acquired and stitched using ZEN (blue edition). Analysis using IMARIS version 7.6 involved creating a surface over the laminin staining, which was masked, allowing a second surface covering each fibre to be created and average fluorescent intensity for each muscle fibre to be collected.

Using values from the negative (no primary antibody) control for each case, non-specific background was subtracted and NDUFB8 and COX-1 intensity was normalised to porin intensity as the mitochondrial mass marker. Control case values then created a “normal population” for which the patient fibres could then be assigned a z-score describing their deviation from the normal population. Statistical analysis is described in more detail by Rocha et al. [24]. Total fibres analysed was dependent on section size and ranged from n = 111 to n = 1883 in patients and controls.

### 2.4 Measurement of porin across the muscle fibre

A total pool of normal (n = 10), low (n = 10) and very low porin (n = 10) fibres were selected to assess porin level across the muscle fibre. The profile tab in ZEN (blue edition) was used to draw a segmenting line across the full muscle fibre and generate a graph of fluorescent intensity in each channel across the fibre.

### 2.5 Mitochondrial mass

To assess mitochondrial mass, labelling of a second marker, succinate dehydrogenase subunit A (SDHA), was compared to porin. Cryosectioned muscle was subject to an immunofluorescence protocol, following a similar protocol as in Section 2.3, but without anti-COXI and replacing anti-NDUFB8 with anti-SDHA. Total fibres analysed across all patients was n = 5565, with fibres per patient ranging from n = 114 to n = 1283 in individual patients and controls.

### 2.6 Analysis of MFM protein aggregates and mitochondrial mass

Muscle sections were co-labelled with antibodies recognising laminin, desmin, myotilin and porin. Following image capture, processing and analysis as in Section 2.3, myofibres containing very low (n = 436), low (n = 338) and normal porin (n = 150) content (across all patients) were analysed in turn and categorised as either being positive or negative for the presence of desmin and myotilin aggregates. Following this, fibres with or without aggregates were plotted against z-score for porin protein level as an indicator of mitochondrial mass.

### 2.7 Laser microdissection and cell lysis

Six patients were selected for mtDNA analysis based on the presence of COX deficient fibres. For each patient four serial muscle sections were cryosectioned. The first and last tissue sections underwent sequential COX/SDH histochemistry and were used to assess deficiency of the second and third sections, which underwent SDH histochemistry only. Sequential single muscle fibres were laser micro-dissected from the second and third tissue section using a PALM system (Zeiss). Fibres from the second tissue section were captured into 15 µl SDS/EDTA/Proteinase K single cell lysis buffer (0.5% SDS, 10 mM EDTA, 5% proteinase K) and incubated for 2 hours at 37 °C to lyse. Fibres from the third were captured into 15 µl Tris/Tween/Proteinase K single cell lysis buffer (0.5M Tris-HCl, 0.5% Tween 20, 1% Proteinase K, pH 8.5) and incubated for 2 hours at 55 °C, followed by 10 minutes at 95 °C to lyse. For each patient 20 single fibres were analysed for both long range PCR and real time PCR.

### 2.8 Long range PCR

Two rounds of PCR were employed to screen isolated muscle fibres for large-scale mtDNA rearrangements: 1 µl cell lysate, 1× PrimeSTAR GXL reaction buffer, 0.2 µM dNTPs, 0.2 µM forward and reverse primers and 0.625 unit polymerase, in a total volume of 50 µl. The first round of PCR amplified a 16179 bp region using primers 2180F (nucleotides m.2180–2209) and 1789R (nucleotides m.1760–1789) and the second round reaction amplified a 16029 bp region using primers 2330F (nucleotides m.2330–2359) and 1789R (nucleotides m.1760–1789) (NC_012920.1). Cycling conditions were: 35 cycles of 10 seconds at 98 °C and 11 minutes at 68 °C. PCR products were separated through a 0.7 % agarose gel with a 1 Kb ladder used to size amplicons.

### 2.9 Triplex real time PCR

A triplex real time PCR assay was used to quantify mtDNA deletion level as described previously [27]. This method quantifies relative levels of *MT-ND4* and *MT-ND1* and D-Loop, using TaqMan chemistry. Primers and TaqMan MGB probes used to detect *MT-ND1*, *MT-ND4* and D-Loop have been previously reported [27]. PCR amplification was completed in a 25 µl reaction in triplicate for each sample, with each plate containing a serial dilution of p7D1 plasmid for standard curve generation, as reported previously [27].

#### 2.9.1 Analysis and statistics

Percentage classification of fibres for NDUFB8, COX-I and porin were compared between the patient group and control group using a Wilcoxon Signed-Rank Test. Spearman's rank correlation (src) was used to assess the relationship between fibre area and complex I, complex IV and porin protein levels.

## 3 Results

### 3.1 Histology and histochemistry

**Fig. 1:**
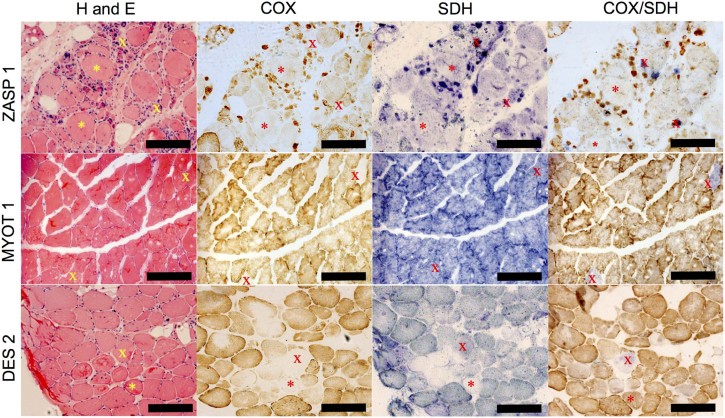
Histochemistry for serial muscle sections. Sections from ZASP 1, MYOT 1 and DES 2 were subject to H&E staining, COX histochemistry, SDH histochemistry and sequential COX/SDH histochemistry. H&E staining demonstrates morphological changes compatible with diagnosis. Many fibres appear COX-deficient but also demonstrate low SDH reactivity, indicative of a reduction in mitochondrial mass. X, COX-deficient fibre, and asterisk (*), mitochondrial depletion fibres. Scale bar 200 µm.

H&E demonstrated a variety of pathological features consistent with a diagnosis of myofibrillar myopathy including: variation in fibre size, rimmed vacuoles, internal nuclei, nuclear bags, basophilic and eosinophilic inclusions, fibre splitting, and necrotic and regenerating fibres. Sequential COX/SDH histochemistry showed a low number of COX-deficient fibres (typically not more than 5%) (Table 2). Furthermore, fibres that were pale for COX activity were also pale for SDH activity, indicating low mitochondrial content (Fig. 1, denoted by asterisk). Many fibres also showed a core-like depletion of mitochondria in the centre and accumulation in the subsarcolemmal region (Fig. 1, DES patient 2).

### 3.2 Clonally-expanded mtDNA deletions

**Fig. 2:**
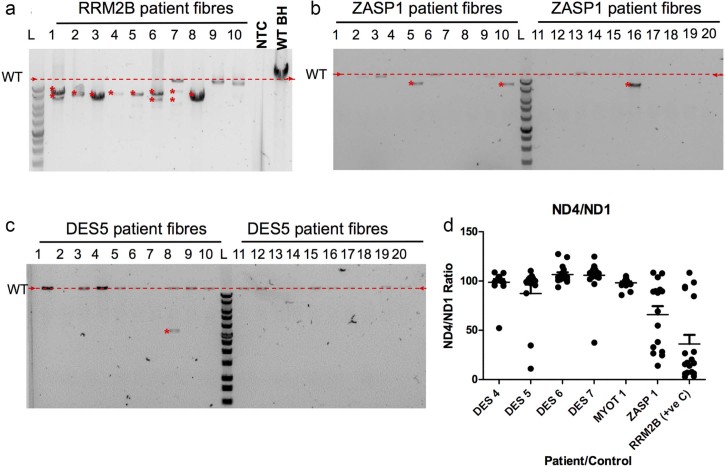
Long range and real time PCR analysis of mtDNA deletions in each patient (n = 20). In long range PCR gels (a–c) red arrows and dashed line indicates size of wild type mtDNA product, red asterisk (*) indicates deletion products. (a) Long range PCR results for 10 single fibre lysates relative to a wild type (WT) blood homogenate, no template control (NTC) and 1 Kb ladder (Promega), multiple and single deletions present due to variants in RRM2B. (b) Long range PCR results for 20 single fibre lysates from ZASP1 demonstrating 3 fibres with a single deletion. (c) Long range PCR results for 20 single fibre lysates from DES 5 demonstrating 1 fibre with an mtDNA deletion. (d) Real time PCR results for DES4, DES5, DES6, DES7, MYOT1 and ZASP1 showing ratios of ND4 to ND1. Lines represent mean and standard error of the mean (SEM).

Long range PCR and real time PCR were used to investigate the presence of clonally-expanded mtDNA deletions in 20 COX-deficient single cell lysates from MFM patients with *DES* (n = 4), *MYOT* (n = 1) and *ZASP* (n = 1) mutations and a positive control (patient with multiple mtDNA deletions caused by genetically inherited pathogenic *RRM2B* variants). For every fibre analysed both long range and real time PCR were performed. Long range PCR analysis of 20 fibres from the patient with *RRM2B* variants demonstrated products of varying size indicative of different sized mtDNA deletions (Fig. 2a). In comparison to the *RRM2B* patient, of the 120 fibres analysed from six MFM patients, an mtDNA deletion was detected in only five fibres (DES4: n = 1, DES5: n = 1 and ZASP1: n = 3). The remaining fibres either had a full-length wild type (WT) product or failed to amplify a product (n = 31) (Fig. 2b and c). Failure to amplify a product is common in long range PCR from single cell lysates. This also occurred in two fibres from the *RRM2B* patient. The percentage of fibres that did not amplify is lower in the *RRM2B* case, likely due to the presence of ragged-red fibres indicative of high mitochondrial mass in the *RRM2B* patient and core-like fibres suggesting low mitochondrial mass in the MFM patients.

Real time PCR analysis showed that the majority of individual COX-deficient myofibres obtained from the *RRM2B* patient muscle contained different levels of mtDNA deletion (Fig. 2d), which was in agreement with long range PCR results (Fig. 2a). Real time PCR also confirmed that only a few MFM muscle fibres contained clonally-expanded mtDNA deletion. Of 120 fibres deletions were found by real time PCR in 12 fibres (DES 4: n = 1, DES5: n = 2, DES7: n = 1 and ZASP1: n = 8), as presented in Fig. 2c and d.

Out of the 120 fibres on which both long range PCR and real time PCR were completed, a deletion was found by both methods in five fibres and a further seven were shown to have a deletion by real time PCR alone. In these seven fibres unfortunately no long range product was amplified. Inclusion of the D-loop allowed confirmation that MFM patient fibres did not contain any deletions affecting *MT-ND1* (data not shown). This was in agreement with long range PCR.

### 3.3 Immunofluorescent analysis of respiratory chain deficiency

Based on the low number of fibres in which COX-deficiency was found by histochemistry and an mtDNA deletion detected, we explored the nature of the respiratory chain protein abundance in more detail. We employed a quantitative immunofluorescence assay to assess porin, NDUFB8 (complex I) and COX-I (complex IV) protein levels.

**Fig. 3:**
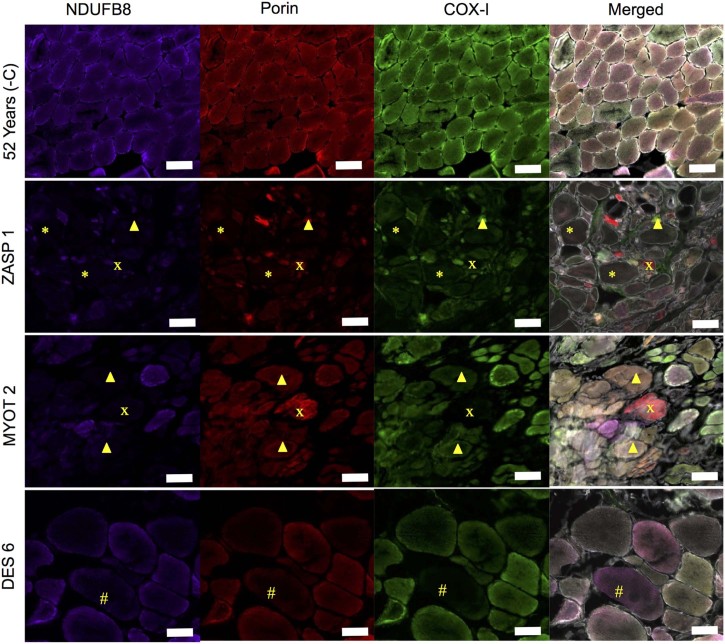
Immunofluorescent analysis of respiratory chain function. Representative immunofluorescence for NDUFB8 (complex I), porin and COX-I (complex IV) in ZASP 1, MYOT 2 and DES 6. Complex I and complex IV deficient fibres, complex IV deficient fibres and complex I deficient fibres can be seen. Most strikingly fibres with low porin, complex I and complex IV can be seen in a number of patients, most pronounced in ZASP1(*). Asterisk (*), low porin complex I and complex IV; Cross, complex I and IV deficient fibres; Arrow head, complex I deficient fibres; and hash, complex IV deficient fibres. Scale bar 100 µm.

**Fig. 4:**
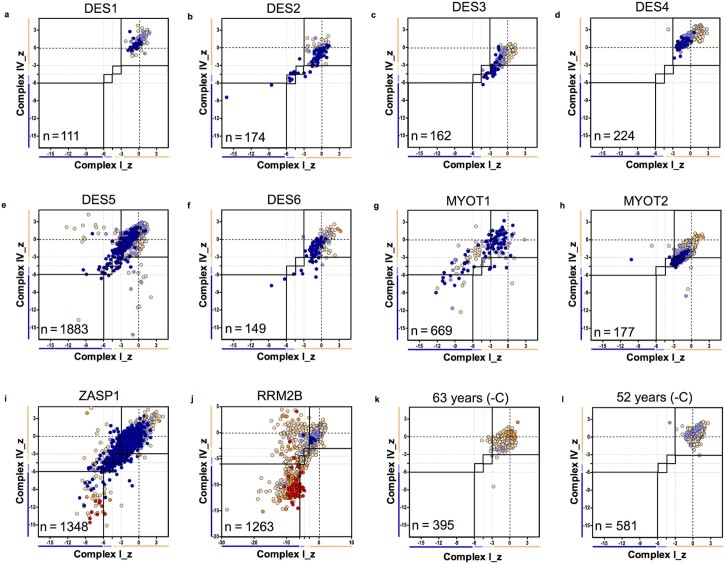
Mitochondrial respiratory chain protein expression profiles. Immunofluorescence results for MFM patients and *RRM2B* patient positive control. Graphs demonstrate distribution of z-scores for complex I and complex IV with each point colour coded for porin category. Number (n) of fibres analysed on each plot. (a) DES1, (b) DES2, (c) DES3, (d) DES4, (e) DES5, (f) DES6, (g) MYOT1, (h) MYOT2, (i) ZASP1, (j) RRM2B, (k) 63 years (negative control) and (l) 52 years (negative control). Each point gives the COX and complex-I z-scores for a single fibre and is colour coded to indicate the porin category of the fibre (very low, dark blue; low, light blue; normal, beige; high, orange; or very high, red). Bars next to the X and Y-axes indicate category complex I or complex IV levels (dark blue, negative; light blue, intermediate negative; light beige, intermediate positive and beige, normal). In addition solid and dashed lines running vertically and horizontally also mark the boundaries between each category of complex I and complex IV.

Immunofluorescent analysis was used to compare the myofibrillar myopathy patients and a patient with compound heterozygous *RRM2B* variants to age matched controls. Immunofluorescent images showed a number of fibres with combined and isolated complex I and complex IV-deficiency, plus fibres with reduced mitochondrial mass (Fig. 3). Respiratory chain protein level profiles for each MFM patient and the RRM2B patient can be found in Fig. 4. A summary of results can be found in Table 3.

Controls aged 52 years and 60 years showed no complex I or complex IV deficiency. In comparison, desmin patients showed some levels of complex I and complex IV deficiency, reaching 6.5% and 4.2% deficient fibres for complex I and complex IV respectively. However, respiratory chain deficiency was low in the majority of desmin patients. Although MYOT1 also had low levels of deficiency, MYOT2 exhibited higher levels ofdeficiency with percentage deficiencies of 45.5% for complex I and 24.7% for complex IV. Finally, the ZASP patient had 11.5% complex I deficient fibres and 9% complex IV deficient fibres.

By comparing these patients with the *RRM2B* patient (32.6% complex I and 23% complex IV deficiency), it is apparent that MYOT2 and ZASP1 exhibit levels of deficiency closer to that of a mitochondrial disease patient.

### 3.4 Reduction in mitochondrial mass

MFM patients showed very low porin fibres between 11% and 51.4%. Low and very low porin fibres are presented by light blue and dark blue circles in the respiratory chain profiles in Fig. 4. There was a significant difference in percentage of normal (p = 0.03), low (p = 0.03) and very low (p = 0.04) porin fibres between patients and controls (tested using a Wilcoxon Signed-Rank Test.) Total fibres were analysed and range of fibres per patient can be found in Section 2.4.

During the image analysis process (using IMARIS), the surfaces created above the fibres did not cover the full area within the laminin membrane staining due to limitations in threshold values used to generate the surfaces for analysis. Previous reports and our findings suggest core-like depletion of mitochondria in the intermyofibrillar regions and aggregation in the subsarcolemmal regions. If this is the case, it is possible that the image analysis process missed the majority of the mitochondria located in the subsarcolemma, explaining the high number of low porin fibres.

In order to test whether we are underestimating the level of porin due to our analysis methods, we analysed the fluorescent signal intensity across a pool of normal, low and very low porin fibres (Supplementary Fig. S1). This demonstrated a reduction in porin fluorescent intensity across the full diameter of the muscle fibre in both low and very low porin fibres when compared to normal fibres.

To further verify mitochondrial mass reduction, porin was compared to SDHA, a nuclearly encoded subunit of complex II. Porin and SDHA immunohistochemistry was carried out using sections from patients and controls. When SDHA is linearly regressed against porin, the r^2^ values suggest a strong positive relationship in the cases with the largest range of porin and SDHA levels. For those cases with a smaller range the relationship was still apparent but the r^2^ value was smaller due to the spread of the data points. This indicates that mitochondrial mass is reduced in a higher percentage of myofibrillar myopathy patient fibres compared to controls (2.6% and 6.4%).

### 3.5 Relation of respiratory chain deficiencies, mitochondrial mass and fibre size

To determine whether levels of porin, complex I and complex IV could be related to fibre atrophy we compared fluorescent intensity for each channel with fibre area. Correlation was examined using Spearman's rank correlation (src) coefficient (summarised in Table 4.). Plotting of muscle fibre area against complex I level (Supplementary Fig. S2) demonstrated a positive correlations in DES5 (src = 0.4389, p < 0.0001), and DES3 (src = 0.4389, p = < 0.0001), a negative correlation in DES6 (src = −0.4817, p < 0.0001). For muscle fibre area and complex IV levels (Supplementary Fig. S3) only very weak to no correlation was found. We also found a negative correlation between muscle fibre area and porin level (Supplementary Fig. S4) in ZASP1 (src = −0.4487, p < 0.0001) and DES6 (src = −0.4583, p < 0.0001). Spearman's rank correlations were also significant for DES2, DES5, DES6, MYOT1 and MYOT2; however, the correlation between porin and muscle fibre area was weak in these patients (Table 4). Porin level plotted against muscle fibre area, for patient ZASP1, in particular, shows that large muscle fibres all have a low porin level whilst smaller muscle fibres have a range of high to low porin. Low Spearman's rank correlations in some patients appear to be due to the smaller range of fibre area.

### 3.6 Association of low porin with protein aggregates

**Fig. 5:**
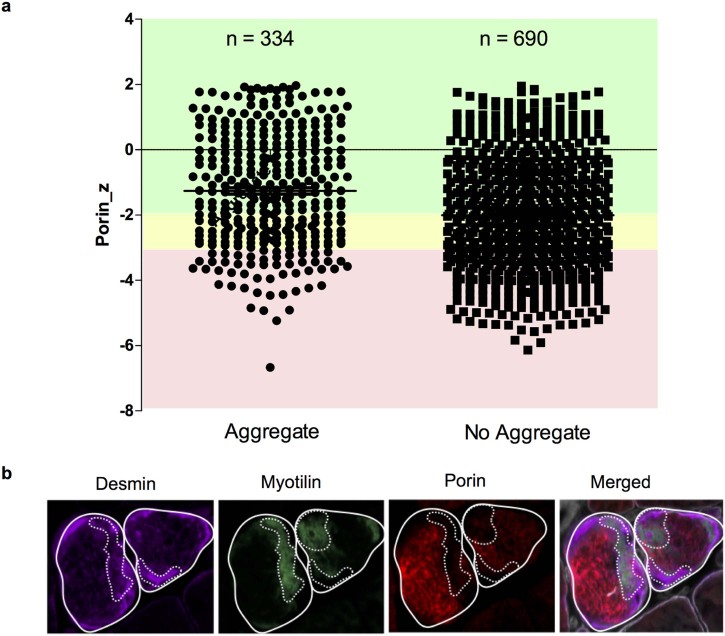
Immunofluorescent analysis of mitochondrial mass and MFM aggregates. ZASP 1 as a representative example of analysis of desmin and myotilin protein aggregation and porin levels. (a) ZASP 1 fibres classified as very low (pink), low (yellow) or normal (green) porin fibres analysed for presence of aggregates. No clear relationship detected. Very low porin fibres accounted for 22.9% of all fibres with aggregates. (b) Immunofluorescent images from ZASP 1 showing individual channel images (desmin, myotilin, porin) and merged. The staining demonstrates a lack of porin in regions with large aggregates (regions within dashed lines).

As protein aggregates are the hallmark of myofibrillar myopathy we hypothesised they may be mechanistically linked to the low porin levels. Analysis of sections co-labelled with antibodies for myotilin, desmin, porin and laminin demonstrated a similar percentage of low and very low porin fibres relative to controls as previously shown. Very low, low and normal porin fibres were analysed for the presence of myotilin and desmin aggregates. This analysis demonstrated no significant relationship between presence of protein aggregates and porin level (Fig. 5b). Furthermore, in fibres with aggregates in ZASP1 (the patient with the highest percentage of low porin fibres), we found that only 22.9% had very low porin levels.

## 4 Discussion

Mitochondrial DNA deletions [20], [21] and respiratory chain deficiency [20], [22], [23] have been shown to accumulate in the skeletal muscle of patients with myofibrillar myopathy. Furthermore changes in mitochondrial positioning have also been found [19]. As such, we aimed to examine a cohort of myofibrillar myopathy patients for the presence of mitochondrial dysfunction, and attempt to understand what correlation, if any, this has with disease pathology.

Here we demonstrate clonally-expanded mtDNA deletions in a small number of fibres from MFM patients by both long range PCR and real time PCR. A further seven fibres were found to have deletions by real time PCR but failed to amplify a product by long range PCR, a common problem for single cell long range PCR especially if mitochondrial content is low as in MFM patient muscle fibres. We conclude that the low levels of mtDNA deletions detected are likely due, at least in part, to the age on top of the disease process. Sporadic deletions are known to form and accumulate in mtDNA of aged muscle [28] and here we find the highest number of deletions in the oldest patient (ZASP1). However, due to the fact that there is only one patient with a ZASP mutation it is difficult to conclude to what extent this is due to age or the genotype.

In fibres where an mtDNA deletion was not detected, it is possible that mtDNA point mutations or mtDNA depletion may be the cause of COX deficiency. In particular, it should be noted that based on both COX/SDH histochemistry and immunofluorescent analysis, COX-deficient fibres are present at lower levels comparable to those with reduced mitochondrial mass. Both quantification of mtDNA copy number and deep sequencing of the mitochondrial genome would be necessary to fully assess this.

Immunofluorescent analysis demonstrated percentage complex I and complex IV deficiency higher than would be expected in age matched controls for four out of six desmin patients, one of two MYOT patients and a ZASP patient.

Most strikingly porin is reduced in all patients, in a significantly high percentage of fibres relative to controls, indicating a reduction in mitochondrial mass. This reduction in mitochondrial mass may simply be due to the depletion of mitochondria in the intermyofibrillar space and aggregation in the subsarcolemmal space previously described in myofibrillar myopathy patients [19], [22], [23]. Analysis of porin fluorescent intensity across a number of normal, low and very low porin fibres demonstrated this is not the case and porin intensity is reduced across the whole fibre. Furthermore, there is no correlation between the presence of desmin and myotilin protein aggregates and the reduction in porin. Throughout the analysis, however, it became apparent that in some fibres where large aggregates are present (Fig. 5c), there is a lack of porin and therefore reduction of mitochondrial mass. In these fibres mitochondria are accumulated in the subsarcolemmal regions or other myofibrillar regions of the fibres, which agrees with previous studies using electron microscopy.

It has been demonstrated that aggregate prone desmin mutations cause a change in Ca^2+^ homeostatic ability, due to lack of appropriate positioning of the mitochondria close to the sarcoplasmic reticulum [29]. Therefore, it may be that aggregate prone mutations in MFM related genes, including *MYOT* and *ZASP* also cause this same effect and therefore lead to increased cytoplasmic Ca^2+^. An increase in cytoplasmic Ca^2+^ concentration in neurons has been found to increased fission and therefore fragment the mitochondrial network [30]. Mitochondrial fusion and interaction of the mitochondrial network are necessary for mitochondrial DNA stability and ability to cope with mtDNA mutation [31]. So, this could explain both the formation and accumulation of mtDNA deletions in some MFM patients and also respiratory chain deficiency. However, whether this could be a relevant mechanism in the reduction of mitochondrial mass or whether a shift in mitochondrial biogenesis and mitophagy might be responsible is unclear.

Comparison of complex I and complex IV levels with muscle fibre cross sectional area yielded mixed relationships, making it hard to determine whether complex I and complex IV levels may be linked to muscle fibre atrophy or hypertrophy. On the other hand, comparison of muscle fibre area with porin levels demonstrated a negative relationship in the majority of patients. The relationship demonstrates that hypertrophic muscle fibres with a greater area typically have a lower level of porin than atrophic fibres with a smaller area, which have more variable levels of porin. This might suggest that mitochondrial mass is not involved in the process of muscle atrophy but may indicate either a lack of mitochondrial biogenesis or increased mitophagy during muscle fibre hypertrophy. Alternatively it could indicate that fibre atrophy is more common in oxidative muscle fibres whilst fibre hypertrophy is more common in glycolytic muscle fibres. It seems most likely that a lack of mitochondrial biogenesis be responsible for the low levels of porin in the hypertrophic muscle fibres; however, further testing would be needed to confirm this.

The low occurrence of mtDNA deletions, lack of relationship between complex I and IV deficiency and muscle fibre cross sectional area and between pathological protein aggregates and mitochondrial dysfunction, found here, are very different to our previous findings in sporadic IBM. In sporadic IBM, approximately 50% of COX-deficient fibres were shown to have one or more large-scale mtDNA rearrangements [32]. Furthermore, inflammatory infiltrates correlated with the extent of the mitochondrial respiratory chain deficiency and muscle fibre atrophy [13]. Therefore, in comparison to IBM where we can conclude mitochondrial dysfunction is pathologically important, and to mitochondrial myopathies where a clear mechanistic link exists between mutations in mtDNA maintenance genes, mtDNA deletions and mitochondrial respiratory chain deficiency, further work needs to be undertaken to determine whether mitochondrial dysfunction is pathologically relevant in MFM. Nevertheless, the decrease in mitochondrial mass and weak relationship between mitochondrial mass and muscle fibre cross sectional area certainly infers that mitochondrial dysfunction may be both mechanistically- and pathologically-relevant.

In conclusion, we have demonstrated clonally-expanded mtDNA deletions in a small number of single cells, similar to previous findings in muscle homogenate DNA from similar patients [20], [21], although do note the increased frequency of these mutations in the oldest patient with a ZASP mutation. Given this and previous work we conclude that mtDNA deletions likely arise due to the combined effect of sarcopenia and disease pathogenesis. We also present evidence that myofibrillar myopathies can manifest depletion of mitochondria and respiratory chain deficiency of varying degrees among patients. Previous reports also report a core-like reduction of mitochondria, something we confirm. Such a depletion of mitochondria may be a contributing factor to overall disease pathogenesis and could be related to muscle fibre hypertrophy.

## Figures and Tables

**Table 1:** Myofibrillar myopathy patient information (n = 9). Patients are organised by gene then age order and all mutation nomenclature uses the primary transcripts for *DES* (NM_001927) and *MYOT* (NM_006790) and for *ZASP* transcript variant 2 (NM_001080114.1).

Patient	Gender	Mutated gene	Mutation	CK	Biopsy site	Age at biopsy	Age at onset (if known)
DES 1	M	*DES*	Het. c.1069G > C, p.(Ala357Pro)	480	Quadriceps	31.2	29
DES 2	M	*DES*	Het. c.1069G > C, p.(Ala357Pro)	NK	NK	32.1	NK
DES 3	M	*DES*	Hom. c.46C > T, p.(Arg16Cys)	450	Quadriceps	33.8	20s
DES 4	F	*DES*	Het. c.735 + 20C > T (MAF = 0.01); c.638C > T, p.(Ala213Val)	NK	Quadriceps	51.3	Early childhood
DES 5	F	*DES*	Het. c.638 C > T, p.(Ala213Val)	74	Quadriceps	53	40s
DES 6	F	*DES*	Het. c.1346A > C , p.(Lys449Thr)	287	NK	64.3	46
MYOT 1	M	*MYOT*	Het. c.179C > G, p.(Ser60Cys)	446	Quadriceps	60.5	55
MYOT 2	M	*MYOT*	c.179C > G, p.(Ser60Cys)	400–500	Tibialis anterior	65.7	NK
ZASP 1	F	*ZASP*	Het. c.494C > T, p.(Ala165Val); Het. c.728C > T, p.(Pro243Leu)	300	Quadriceps	69.6	60s

DES: desmin; MYOT: myotilin; Hom.: homozygous; Het.: heterozygous; MAF: maternal allele frequency; CK: creatine kinase; NK: not known.

**Table 2:** COX deficiency counts for myofibrillar myopathy patients based on sequential COX/SDH histochemistry.

Patient	Total fibres	COX-positive	Intermediate- positive	Intermediate-negative	COX negative
DES 1	494	94.3	4.45	1.01	0.202
DES 2	611	87.7	10.3	1.80	0.164
DES 3	127	81.9	18.1	0.0	0.0
DES 4	863	95.6	4.06	0.348	0.0
DES 5	1086	93.6	5.06	0.829	0.552
DES 6	248	45.16	39.1	12.9	2.82
MYOT 1	848	96.6	2.48	0.943	0.0
MYOT 2	697	91.1	5.67	3.73	0.0
ZASP 1	835	99.4	0.479	0.120	0.0

**Table 3:** Immunofluorescent analysis of respiratory chain protein expression. Summary of percentage of fibres classified as complex I and complex IV negative or deficient (negative and intermediate categories). Controls and patients are organised in age order for easy comparison.

Patient	Age/years	Complex I negative (%)	Complex I deficient (%)	Complex IV negative (%)	Complex IV deficient (%)	Total fibres analysed
DES 1	31.2	0.00	0.00	0.00	0.00	111
DES 2	32.1	0.12	6.48	0.12	3.62	174
DES 3	33.8	0.00	0.00	0.58	4.18	162
DES 4	51.3	0.00	0.00	0.00	0.00	224
52 Years (-ve C)	52.0	0.00	0.00	0.00	0.00	581
DES 5	53.0	0.79	1.41	0.8	1.63	1883
60 Years (-ve C)	60.0	0.00	0.73	0.07	0.07	395
MYOT 1	60.5	0.18	0.18	0.12	0.44	669
DES 6	64.3	1.91	2.50	3.52	4.99	149
MYOT 2	65.7	38.5	45.51	13.57	24.73	177
ZASP 1	69.6	6.43	11.47	5.34	8.96	1348

**Table 4:** Summary of Spearman's rank correlation (src) analysis of muscle fibre are area against levels of porin, complex I and complex IV.

Patient	Porin		Complex I		Complex IV	
	src	p value	src	p value	src	p value
DES 1	−0.0564	0.5546	0.1389	0.144	0.09819	0.303
DES 2	−0.2492	0.0009	0.1161	0.1261	−0.1738	0.0214
DES 3	0.3776	<0.0001	0.4389	<0.0001	0.2808	0.0003
DES 4	0.06009	0.3707	0.1249	0.0619	0.05067	0.4504
DES 5	−0.1733	<0.0001	0.26	<0.0001	0.2933	<0.0001
DES 6	−0.4583	<0.0001	−0.4817	<0.0001	−0.3073	0.0001
MYOT 1	−0.2093	<0.0001	−0.09169	0.0187	−0.2006	<0.0001
MYOT 2	0.1302	0.1064	0.2428	0.0023	0.0483	0.5506
ZASP 1	−0.4487	<0.0001	−0.1716	<0.0001	−0.1634	<0.0001

## Appendix Supplementary material

Fig. S1 Across fibre porin analysis. Examples of very low and normal porin fibres and the fluorescent intensity profiles generated across a bisecting line. Measurements were completed in ZEN and graphs generated with GraphPad Prism. Fig. S1

Fig. S2 Complex I z score (Complex I_z) against fibre area z score (Area_z) for: (a) DES1, (b) DES2, (c) DES3, (d) DES4, (e) DES5, (f) DES6, (g), MYOT1, (h) MYOT2 and (i) ZASP1. Spearman rank correlation and p values in Table 4. Fig. S2

Fig. S3 Plots of Complex IV z score (Complex IV_z) against fibre area z score (Area_z) for: (a) DES1, (b) DES2, (c) DES3, (d) DES4, (e) DES5, (f) DES6, (g), MYOT1, (h) MYOT2 and (i) ZASP1. Spearman rank correlation and p values in Table 4. Fig. S3

Fig. S4 Porin z score (Porin_z) against fibre area z score (Area_z) for: (a) DES1, (b) DES2, (c) DES3, (d) DES4, (e) DES5, (f) DES6, (g), MYOT1, (h) MYOT2 and (i) ZASP1. Spearman rank correlation and p values in Table 4. Fig. S4

## References

[1] Goldfarb L.G., Park K.Y., Cervenakova L. (1998). Missense mutations in desmin associated with familial cardiac and skeletal myopathy. Nat Genet.

[2] Vicart P., Caron A., Guicheney P. (1998). A missense mutation in the alphaB-crystallin chaperone gene causes a desmin-related myopathy. Nat Genet.

[3] Selcen D., Engel A.G. (2004). Mutations in myotilin cause myofibrillar myopathy. Neurology.

[4] Vorgerd M., van der Ven P.F., Bruchertseifer V. (2005). A mutation in the dimerization domain of filamin c causes a novel type of autosomal dominant myofibrillar myopathy. Am J Hum Genet.

[5] Selcen D., Engel A.G. (2005). Mutations in ZASP define a novel form of muscular dystrophy in humans. Ann Neurol.

[6] Selcen D., Muntoni F., Burton B.K. (2009). Mutation in BAG3 causes severe dominant childhood muscular dystrophy. Ann Neurol.

[7] Oldfors A., Larsson N.G., Lindberg C., Holme E. (1993). Mitochondrial DNA deletions in inclusion body myositis. Brain.

[8] Oldfors A., Moslemi A.R., Fyhr I.M., Holme E., Larsson N.G., Lindberg C. (1995). Mitochondrial DNA deletions in muscle fibers in inclusion body myositis. J Neuropathol Exp Neurol.

[9] Santorelli F.M., Sciacco M., Tanji K. (1996). Multiple mitochondrial DNA deletions in sporadic inclusion body myositis: a study of 56 patients. Ann Neurol.

[10] Moslemi A.R., Lindberg C., Oldfors A. (1997). Analysis of multiple mitochondrial DNA deletions in inclusion body myositis. Hum Mutat.

[11] Askanas V., Engel W.K. (2006). Inclusion-body myositis: a myodegenerative conformational disorder associated with Abeta, protein misfolding, and proteasome inhibition. Neurology.

[12] Oldfors A., Moslemi A.R., Jonasson L., Ohlsson M., Kollberg G., Lindberg C. (2006). Mitochondrial abnormalities in inclusion-body myositis. Neurology.

[13] Rygiel K.A., Miller J., Grady J.P., Rocha M.C., Taylor R.W., Turnbull D.M. (2015). Mitochondrial and inflammatory changes in sporadic inclusion body myositis. Neuropathol Appl Neurobiol.

[14] Chariot P., Ruet E., Authier F.J., Labes D., Poron F., Gherardi R. (1996). Cytochrome c oxidase deficiencies in the muscle of patients with inflammatory myopathies. Acta Neuropathol.

[15] Askanas V., Engel W.K., Nogalska A. (2012). Pathogenic considerations in sporadic inclusion-body myositis, a degenerative muscle disease associated with aging and abnormalities of myoproteostasis. J Neuropathol Exp Neurol.

[16] Bender A., Krishnan K.J., Morris C.M. (2006). High levels of mitochondrial DNA deletions in substantia nigra neurons in aging and Parkinson disease. Nat Genet.

[17] Greaves L.C., Yu-Wai-Man P., Blakely E.L. (2010). Mitochondrial DNA defects and selective extraocular muscle involvement in CPEO. Invest Ophthalmol Vis Sci.

[18] Grunewald A., Rygiel K.A., Hepplewhite P.D., Morris C.M., Picard M., Turnbull D.M. (2016). Mitochondrial DNA depletion in respiratory chain-deficient Parkinson disease neurons. Ann Neurol.

[19] Claeys K.G., Fardeau M., Schroder R. (2008). Electron microscopy in myofibrillar myopathies reveals clues to the mutated gene. Neuromuscul Disord.

[20] Dold T., Reimann J., Zsurka G., Kunz W.S., Kornblum C. (2012). On mitochondrial function and genome integrity in myofibrillar myopathies. Neuromuscul Disord.

[21] Joshi P.R., Hauburger A., Kley R. (2014). Mitochondrial abnormalities in myofibrillar myopathies. Clin Neuropathol.

[22] Henderson M., De Waele L., Hudson J. (2013). Recessive desmin-null muscular dystrophy with central nuclei and mitochondrial abnormalities. Acta Neuropathol.

[23] Schroder R., Goudeau B., Simon M.C. (2003). On noxious desmin: functional effects of a novel heterozygous desmin insertion mutation on the extrasarcomeric desmin cytoskeleton and mitochondria. Hum Mol Genet.

[24] Rocha M.C., Grady J.P., Grunewald A. (2015). A novel immunofluorescent assay to investigate oxidative phosphorylation deficiency in mitochondrial myopathy: understanding mechanisms and improving diagnosis. Sci Rep.

[25] Pitceathly R.D., Smith C., Fratter C. (2012). Adults with RRM2B-related mitochondrial disease have distinct clinical and molecular characteristics. Brain.

[26] Taylor R.W., Barron M.J., Borthwick G.M. (2003). Mitochondrial DNA mutations in human colonic crypt stem cells. J Clin Invest.

[27] Rygiel K.A., Grady J.P., Taylor R.W., Tuppen H.A.L., Turnbull D.M. (2015). Triplex real-time PCR – an improved method to detect a wide spectrum of mitochondrial DNA deletions in single cells. Sci Rep.

[28] McKenzie D., Bua E., McKiernan S., Cao Z., Aiken J.M. (2002). Mitochondrial DNA deletion mutations: a causal role in sarcopenia. Eur J Biochem.

[29] Smolina N., Bruton J., Sjoberg G., Kostareva A., Sejersen T. (2014). Aggregate-prone desmin mutations impair mitochondrial calcium uptake in primary myotubes. Cell Calcium.

[30] Han X.J., Lu Y.F., Li S.A. (2008). CaM kinase I alpha-induced phosphorylation of Drp1 regulates mitochondrial morphology. J Cell Biol.

[31] Chen H., Vermulst M., Wang Y.E. (2010). Mitochondrial fusion is required for mtDNA stability in skeletal muscle and tolerance of mtDNA mutations. Cell.

[32] Rygiel K.A., Tuppen H.A., Grady J.P. (2016). Complex mitochondrial DNA rearrangements in individual cells from patients with sporadic inclusion body myositis. Nucleic Acids Res.

