# Supplementary figures and images for: Mitochondrial dysfunction in myofibrillar myopathy
Source: Neuromuscul Disord. 2016 Oct;26(10):691–701. doi: 10.1016/j.nmd.2016.08.004 (PMC5066370; doi:10.1016/j.nmd.2016.08.004)

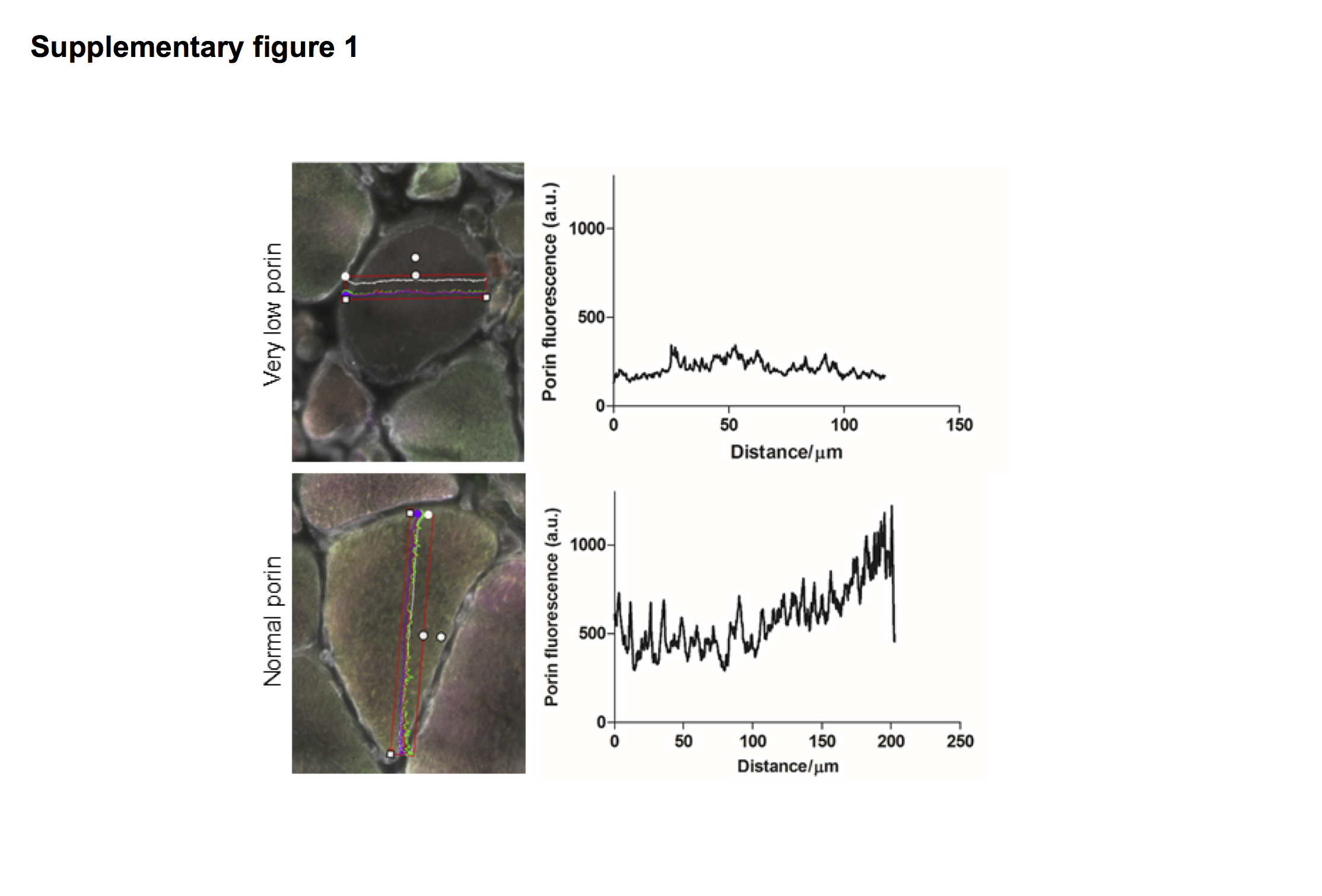

Supplement: Fig. S1 — Across fibre porin analysis. Examples of very low and normal porin fibres and the fluorescent intensity profiles generated across a bisecting line. Measurements were completed in ZEN and graphs generated with GraphPad Prism. [file mmc1.zip › mmc1.tiff]

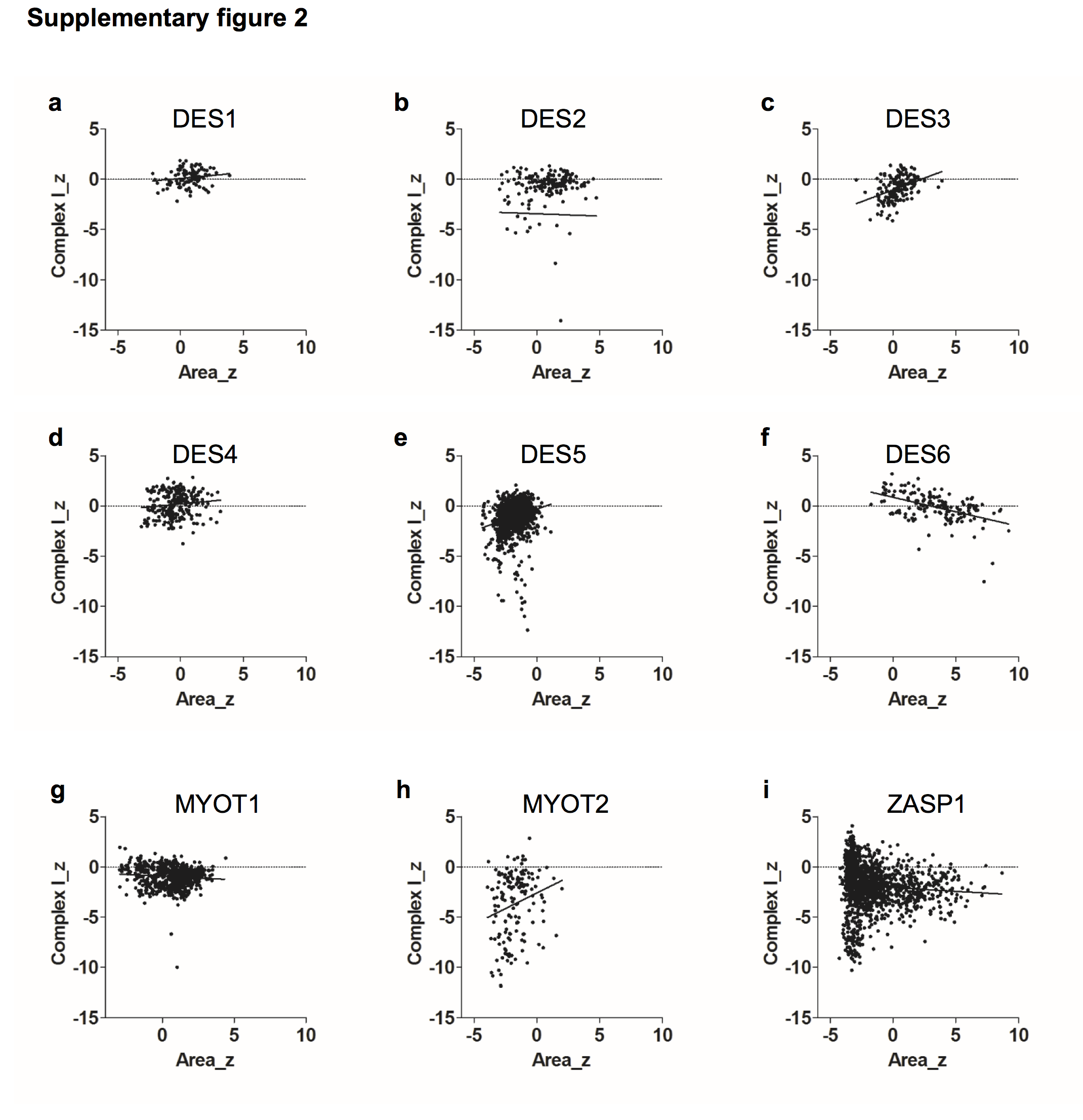

Supplement: Fig. S2 — Complex I z score (Complex I_z) against fibre area z score (Area_z) for: (a) DES1, (b) DES2, (c) DES3, (d) DES4, (e) DES5, (f) DES6, (g), MYOT1, (h) MYOT2 and (i) ZASP1. Spearman rank correlation and p values in Table 4. [file mmc2.zip › mmc2.tiff]

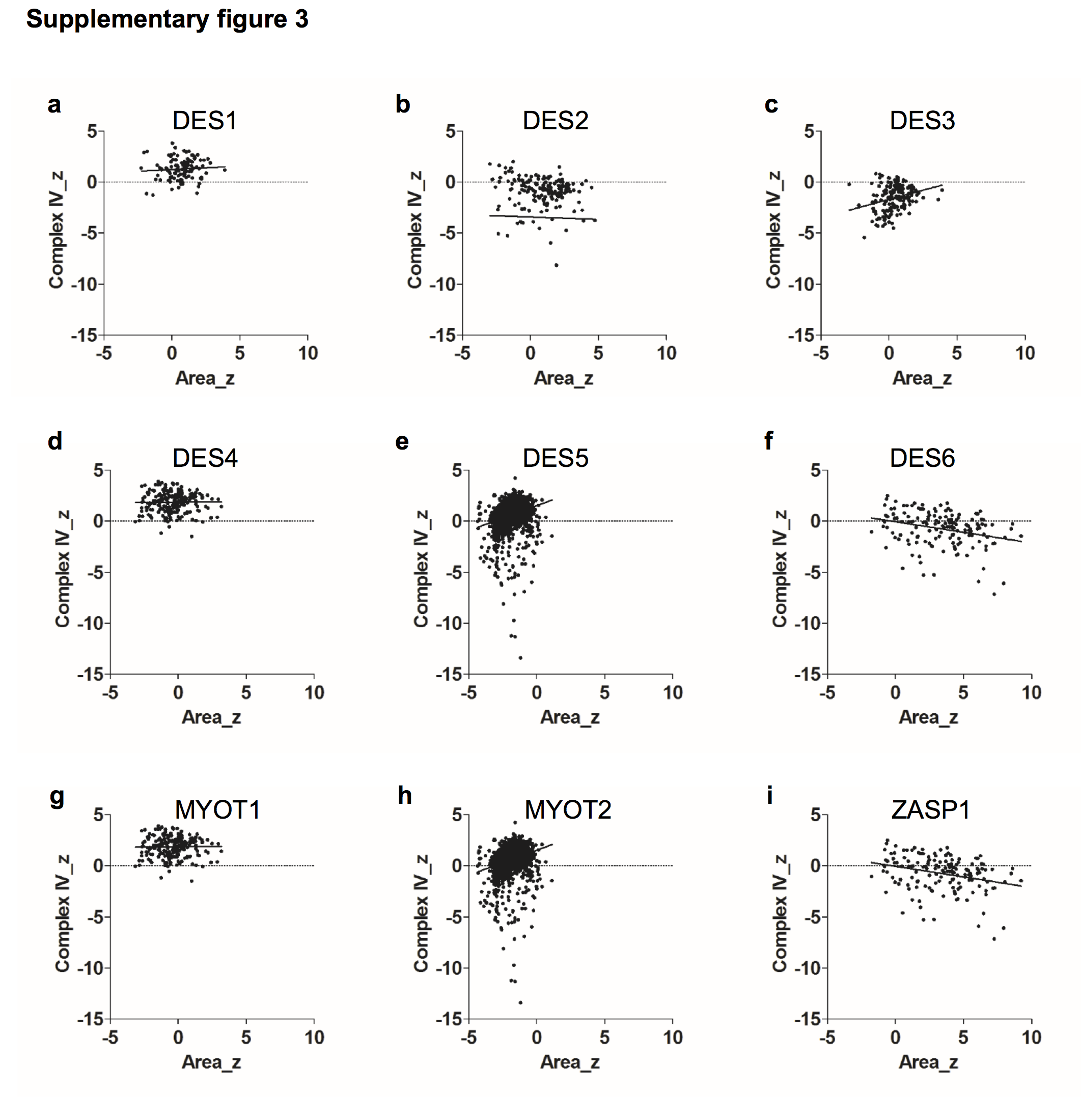

Supplement: Fig. S3 — Plots of Complex IV z score (Complex IV_z) against fibre area z score (Area_z) for: (a) DES1, (b) DES2, (c) DES3, (d) DES4, (e) DES5, (f) DES6, (g), MYOT1, (h) MYOT2 and (i) ZASP1. Spearman rank correlation and p values in Table 4. [file mmc3.zip › mmc3.tiff]

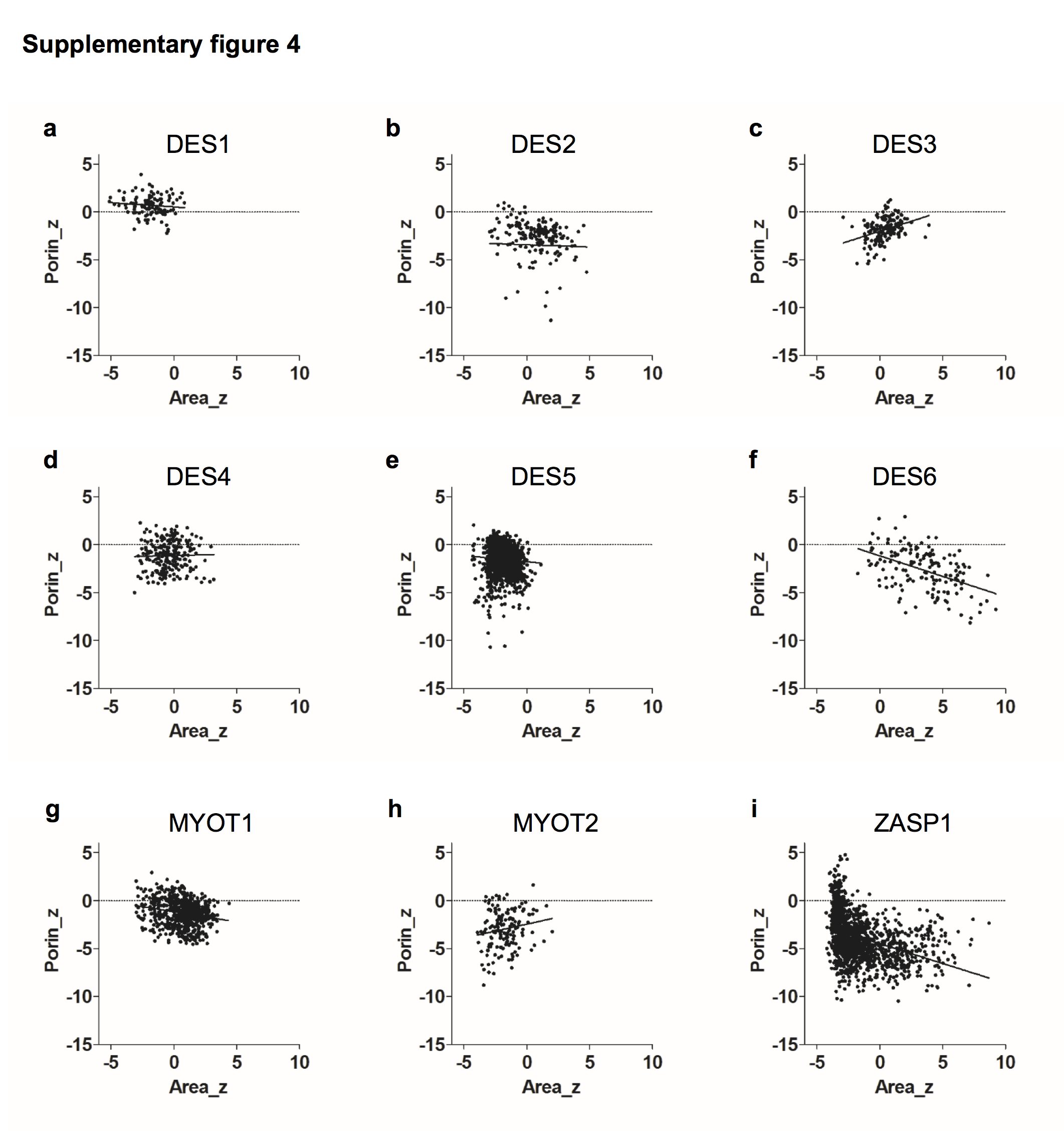

Supplement: Fig. S4 — Porin z score (Porin_z) against fibre area z score (Area_z) for: (a) DES1, (b) DES2, (c) DES3, (d) DES4, (e) DES5, (f) DES6, (g), MYOT1, (h) MYOT2 and (i) ZASP1. Spearman rank correlation and p values in Table 4. [file mmc4.zip › mmc4.tiff]
